# Correction: Construction of an autocatalytic reaction cycle in neutral medium for synthesis of life-sustaining sugars

**DOI:** 10.1039/d5sc90052b

**Published:** 2025-02-24

**Authors:** Hiro Tabata, Genta Chikatani, Hiroaki Nishijima, Takashi Harada, Rika Miyake, Souichiro Kato, Kensuke Igarashi, Yoshiharu Mukouyama, Soichi Shirai, Minoru Waki, Yoko Hase, Shuji Nakanishi

**Affiliations:** a Research Center for Solar Energy Chemistry, Graduate School of Engineering Science, Osaka University Toyonaka Osaka 560-8531 Japan nakanishi.shuji.es@osaka-u.ac.jp; b Bioproduction Research Institute, National Institute of Advanced Industrial Science and Technology (AIST) 2-17-2-1, Tsukisamu higashi, Toyohira Sapporo 062-8517 Japan; c Division of Science, College of Science and Engineering, Tokyo Denki University Hatoyama Saitama 350-0394 Japan; d Toyota Central R&D Labs, Inc. 41-1 Yokomichi Nagakute Aichi 480-1192 Japan y-hase@mosk.tytlabs.co.jp; e Innovative Catalysis Science Division, Institute for Open and Transdisciplinary Research Initiatives (ICS-OTRI), Osaka University Suita Osaka 565-0871 Japan

## Abstract

Correction for ‘Construction of an autocatalytic reaction cycle in neutral medium for synthesis of life-sustaining sugars’ by Hiro Tabata *et al.*, *Chem. Sci.*, 2023, **14**, 13475–13484, https://doi.org/10.1039/D3SC03377E.

The authors regret that there are a number of typographical errors in the article, as detailed here.

The text reading “The main text of the article should appear here with headings as appropriate.” near the bottom of the second column on page 13477 should be deleted.

Part (d) of the caption for Fig. 3 should read as follows: “(d) Proposed reaction scheme for r-2 → r-6 in which a sodium ion and a Brønsted base work concertedly on HCHO and **C2a** → **C4a**. Calculated free energy diagram for r-6 catalyzed by NaOH (black) or Na_2_WO_4_ (brown) in water”.

The text “LVBE transformations” near the bottom of the first column on page 13478 should read “LVBE → LBVE transformations”.

The Royal Society of Chemistry apologises for these errors and any consequent inconvenience to authors and readers.

